# Understanding the telehealth experience of care by people with ILD during the COVID-19 pandemic: what have we learnt?

**DOI:** 10.1186/s12890-023-02396-6

**Published:** 2023-04-06

**Authors:** Gabriella Tikellis, Tamera Corte, Ian N. Glaspole, Nicole Goh, Yet H. Khor, Jeremy Wrobel, Karen Symons, Lisa Fuhrmeister, Laura Glenn, Shiji Chirayath, Lauren Troy, Anne E. Holland

**Affiliations:** 1 Respiratory Research@Alfred, Central Clinical School, Monash University, Melbourne, VIC Australia; 2 NHMRC Centre of Research Excellence in Pulmonary Fibrosis, Sydney, Australia; 3 Department of Allergy, Clinical Immunology and Respiratory Medicine, Monash University, Melbourne, Australia; 4 Royal Prince Alfred Hospital, Sydney, Australia; 5 Central Clinical School, The University of Sydney, Sydney, NSW Australia; 6 Department of Respiratory Medicine, Alfred Health, Melbourne, VIC Australia; 7 Department of Respiratory and Sleep Medicine, Austin Health, Melbourne, VIC Australia; 8 Institute for Breathing and Sleep, Melbourne, Australia; 9 Faculty of Medicine, University of Melbourne, Melbourne, VIC Australia; 10 Fiona Stanley Hospital, Perth, WA Australia; 11 School of Medicine, University of Notre Dame, Fremantle, WA Australia; 12 Department of Physiotherapy, Alfred Health, Melbourne, Australia

**Keywords:** Interstitial lung disease, Pulmonary fibrosis, COVID-19 pandemic, Healthcare experiences

## Abstract

**Introduction:**

The COVID-19 pandemic resulted in a rapid transformation of health services. This study aimed to understand the experiences of healthcare by people with interstitial lung disease (ILD), to inform future service delivery.

**Methods:**

Four specialist clinics in tertiary centres in Australia (Victoria:2 sites; New South Wales: 1 site; Western Australia: 1 site) recruited patients with ILD during an 8-week period from March 2021. Participants completed a COVID-specific questionnaire focused on health-related experiences during 2020.

**Results:**

Ninety nine (65% of 153) participants completed the questionnaire. 47% had idiopathic pulmonary fibrosis or connective tissue disease-associated ILD, 62% were female and the average age was 66 years. Whilst 56% rated their overall health in 2020 as the same as months prior, 38% indicated a worsening in health attributed to reduced physical activity and fear of contracting the virus. Access to healthcare professionals was ‘good’ in 61%, and ‘fair-to-poor’ for 37% due to missed respiratory assessments, with telehealth (mainly telephone) being perceived as less effective. 89% had contact with respiratory physicians, 68% with general practitioners, predominantly via telephone, with few video consultations. High satisfaction with care was reported by 78%, with lower satisfaction attributed to delays in assessments, disruption to usual services such as pulmonary rehabilitation, and dissatisfaction with telehealth.

**Conclusion:**

People with ILD were generally satisfied with their care during 2020, however reduced access to healthcare professionals was challenging for those experiencing a deterioration in health. Telehealth was largely well received but did not always meet the needs of people with ILD particularly when unwell.

**Supplementary Information:**

The online version contains supplementary material available at 10.1186/s12890-023-02396-6.

## Introduction

Interstitial lung diseases (ILD) include life-threatening fibrotic diseases associated with a high symptom burden, often a dismal prognosis and frequently a progressive decline in lung function that requires regular monitoring for optimal management [1]. Aside from pharmacological therapies including anti-fibrotic medications, there is growing evidence to support the use of non-pharmacological therapies such as pulmonary rehabilitation (PR) and supplemental oxygen in managing symptoms given a cure remains elusive [2]. Crises such as the COVID-19 pandemic impose prolonged and extreme demands on healthcare systems. In Australia and around the world, the pandemic has resulted in a rapid transformation of society and health services, necessitating sudden changes in access to, and delivery of, healthcare for people with ILD.

**Fig. 1:**
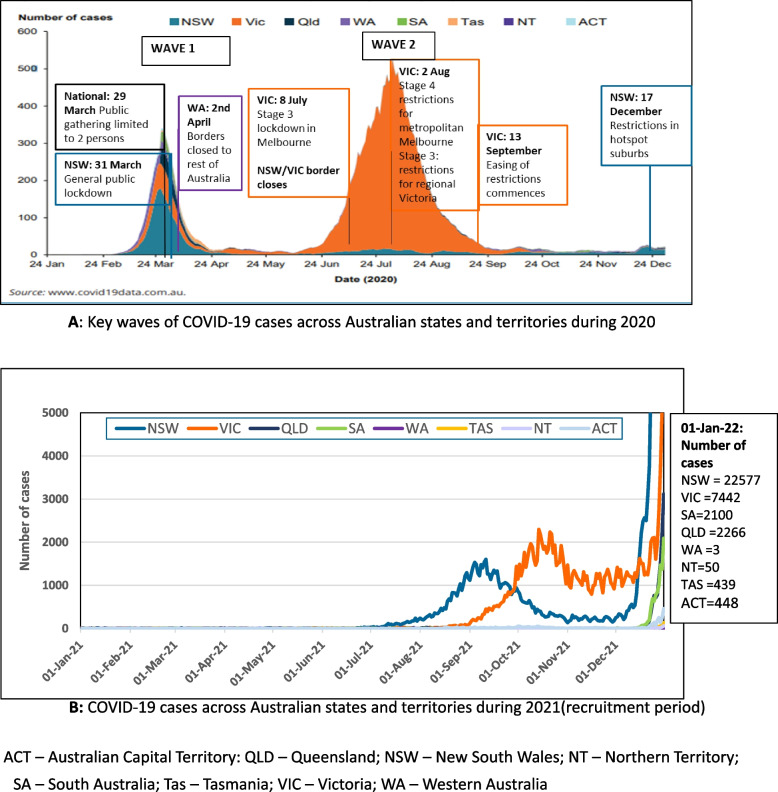
**A** Key waves of COVID-19 cases across Australian states and territories during 2020. **B** COVID-19 cases across Australian states and territories during 2021 (recruitment period). ACT – Australian Capital Territory; QLD – Queensland; NSW – New South Wales; NT – Northern Territory; SA – South Australia;<br/> Tas – Tasmania; VIC – Victoria; WA – Western Australia

The first case of COVID-19 in Australia was detected on 25 January 2020 with around 28,500 cases reported by the end of 2020. Outbreaks varied in time and location around the country with two main ‘waves’ occurring in March/April and June to September 2020 (Fig. 1A) and a further wave mid-late 2021 (Fig. 1B) [3]. The first peak affected all states and territories with New South Wales (NSW) having the largest number of cases, whilst the second peak was almost completely in Victoria. As a result, fundamental health services for people with ILD including lung function assessments, imaging, and access to medical consultations and supportive care services ceased, were significantly reduced, or conducted via telehealth from early March 2020.

Due to their pre-existing lung disease and treatment often involving immunosuppressive agents, people with ILD are generally more susceptible to infections and shown to be at greater risk of mortality from COVID-19, compared to those without ILD [4, 5]. Several cross-sectional surveys conducted early in 2020 reported that markers of emotional distress and negative impacts on daily life were common in patients with ILD and higher than the general public. In addition, a key concern for patients was the ability to safely access hospital services [6, 7].

The impact of the pandemic during 2020 on the accessibility of healthcare for people with ILD has not been thoroughly explored. Our aim was to understand the experiences of care in people with ILD during the rapid transformation to telehealth to inform the design of service delivery moving forward.

## Methods

### Study population

Respiratory physicians recruited eligible participants over an eight-week period from those attending in-person clinics from four ILD clinics located within metropolitan tertiary centres: two sites in Victoria (March to April 2021), one in NSW (May to June 2021) and one in Western Australia (WA) (August to September 2021). Eligibility criteria included a diagnosis of ILD prior to 2020, an existing outpatient at the ILD clinic at each respective site, aged 18 years or older and able to provide informed consent.

Demographic and clinical data were extracted from medical records and used to characterise participants.

### Questionnaires

Participants completed three self-administered questionnaires online or a paper copy was mailed out.

A 19-item, COVID-specific questionnaire was developed in consultation with healthcare professionals (HCPs) and people living with ILD to collect information on the impact of the COVID-19 pandemic on the health care experiences of people with ILD during 2020 (Additional file 1). The questionnaire was one component of a larger study investigating the impact of the pandemic on ILD. Health-related quality of life (HRQoL) was assessed using The King's Brief Interstitial Lung Disease (K-BILD) and shortness of breath using The University of California San Diego Shortness of Breath questionnaire (UCSD-SOB) [8, 9]. Higher total K-BILD scores indicated a better HRQoL whilst higher scores in the UCSD-SOB questionnaire indicated greater dyspnoea.

### Analyses

Clinical data and questionnaire items were analysed descriptively as n (% of total) or mean and standard deviation. For clinical data extraction, dates when each clinic changed from face-to-face to telehealth care were determined for each site: 01 April 2020 for site 1 in Victoria, 25 March 2020 for site 2 in Victoria, 9 March 2020 for the NSW site and 16 March 2020 for the WA site. Open text comments were analysed using thematic analysis to identify common themes [10].

## Results

A total of 99 (65% of 153 eligible participants approached to participate) participants completed the COVID-19 specific questionnaire. However, only 85 respondents provided identifying information to allow their clinical data to be extracted and thus, form the basis of these analyses. Detailed responses are available in Additional file 2.

Characteristics of the 85 respondents are described in Table 1 and indicate a wide representation of demographics, ILD type and severity. On average, participants were 66 years of age, 47% had either IPF or CTD-ILD, 74% were on ILD medications (either antifibrotics or immunosuppressants) and 21% had undertaken a PR program within the last 12 months. Whilst 41% were tested for COVID, none returned a positive result and 28% reported a hospital admission with infection being the reason in 33%.

**Table 1:** Characteristics of the 85 participants who completed the COVID-19 specific questionnaire and provided clinical data

**Characteristic**	**N**	
**Site,** n (%)	85	
- Victoria site 1		20 (23)
- Victoria site 2		20 (23)
- New South Wales (NSW)		16 (19)
- Western Australia (WA)		29 (34)
**Age**, *years (mean* ± *SD, Range)*	85	66.1 ± 10.9; Range 29 – 87
**Gender**, female *n (%)*	85	53 (62)
**Smoking status** *n (%)*	80	
Never		29 (36)
Former		49 (61)
Current		2 (3)
**BMI**, kg/m^2^ *(mean* ± *SD, Range)*	72	29.7 ± 6.0; Range 20.5 – 49.0
**ILD type** *n (%)*	82	
- CTD-ILD		19 (23)
- HP		6 (7)
- IPF		20 (24)
- NSIP		14 (17)
- Sarcoidosis		7 (9)
- Scleroderma		1 (1)
- Unclassifiable ILD		7 (9)
- Other		8 (10)
**Years since diagnosed with ILD,** *(mean* ± *SD, Range)*	79	5.4 ± 4.2; Range 0.5 – 25
**ILD medications use,** *yes, n (%)*	85	63 (74) *(more than one option allowed)*
*Type*		
- Methotrexate		5 (8)
- Mycophenolate		26 (41)
- Nintedanib		12 (19)
- Pirfenidone		8 (13)
- Prednisolone		17 (27)
- Rituximab		1 (2)
**Oxygen use** – currently, *yes, n (%)*	81	14 (17)
**Pulmonary rehabilitation in last 12 months**, *Yes, n (%)*	80	17 (21)
**Lung function assessments**		
** 6MWT**, m^a^ *(mean* ± *SD, Range)*	61	459.7 ± 132.6; Range 150 –774
** FVC %pred** *(mean* ± *SD, Range)*^a^	69	75.4 ± 16.7; Range 34 – 116
** DLCO %pred** *(mean* ± *SD, Range)*^a^	71	58.1 ± 17.0; Range 21 – 102
**Validated questionnaire scores**		
**K-BILD transformed score**, *(mean* ± *SD, Range)*	85	55.8 ± 12.9; Range 27.2 – 100
**USCD-SOB score**, *(mean* ± *SD, Range)*	85	43.6 ± 25.0; Range 2 – 100
**Comorbidities**		
*Main comorbidities*	85	*n (%)*
- Autoimmune rheumatological disease		12 (14%)
- Chronic kidney disease		11 (13%)
- Diabetes mellitus		9 (11%)
- Haematological cancer *(within last 5 years)*		0 (0%)
- Heart disease		5 (6%)
- Non-haematological cancer* (within last 12 months)*		1 (1%)
**Infections and hospitalization in 2020**		
Tested for COVID-19 infection, *yes* n(%)	85	35 (41)
- *Number of positive tests*		0 (0)
Lung infection, *yes* n(%)	85	19 (22)
Hospital admission^b^, *yes* n(%)	85	24 (28)
*Main reason for admission*		
- Infection		8 (33)
- Oxygen desaturation/dyspnoea		5 (21)
- Lung biopsy		3 (13)
- Kidney-related		3 (13)
- Other		5 (21)

*CTD-ILD *Connective tissue disease-ILD, *HP *hypersensitive pneumonitis, *IPF *idiopathic pulmonary fibrosis, *NSIP *Nonspecific interstitial pneumonia*, 6MWT *6 min walking test*, FVC% pred *forced vital capacity % predicted*, DLCO% pred *diffusing capacity for carbon monoxide % predicted, *K-BILD *The King’s Brief Interstitial Lung Disease*, USCD-SOB *The University of California San Diego Shortness of Breath

^a^Refers to last result prior to date when face-to-face clinic consultations changed to telehealth

^b^Hospital admission defined as staying one or more nights

During 2020, 89% reported having contact with their respiratory physician with 67% of consultations being conducted by telephone, 57% in person and 8% using videoconferencing. The main reasons reported for making contact were symptom deterioration or regular follow up appointments. Sixty six percent reported contact with their GP of which 79% of consultations were conducted by telephone, 75% in person and 4% by videoconferencing. Reasons for contact included routine/regular follow-up appointment, need for prescriptions or referrals and for vaccinations. Nurses were a contact point for 26% of respondents, with experiencing shortness of breath a common reason for contact. Other HCPs contacted included rheumatologists, immunologists, psychologists, oncologists, sleep physicians and urologists. Comparing those who reported having contact with any health professional only by telemedicine (phone or video, *n* = 22) with those who had contact by in-person visits, phone and/or video (*n* = 63), we found those who accessed telemedicine only, had on average better lung health as indicated by greater average 6MWT distance (485.60 ± (SD)140.10 m vs 451.22 ± 130.57), better FVC% predicted (79.39 ± 19.70 vs 73.78 ± 15.16) and DLCO% predicted (62.65 ± 15.82 vs 56.33 ± 17.21) and lower UCSD-SOB score indicating less dyspnoea (36.50 ± 5.64 vs 46.03 ± 24.21). However, differences were not statistically significant.

**Fig. 2:**
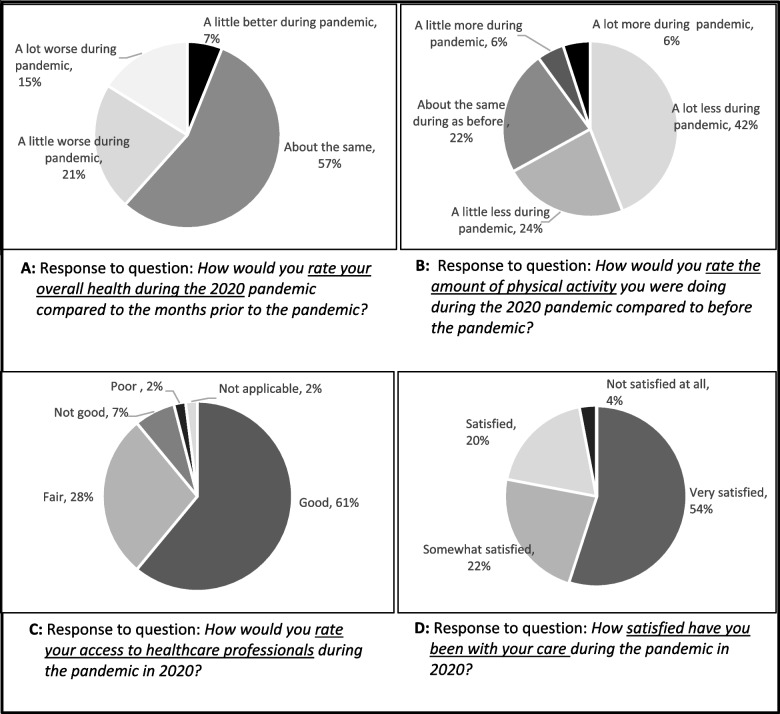
Responses to questionnaire items on overall health during 2020 (Panel 2**A**); amount of physical activity undertaken during 2020 (Panel 2**B**); access to healthcare professionals during 2020 (Panel 2**C**) and satisfaction with care during 2020 (Panel 2**D**). ACT – Australian Capital Territory; QLD – Queensland; NSW – New South Wales; NT – Northern Territory; SA – South Australia;<br/> Tas – Tasmania; VIC – Victoria; WA – Western Australia

Most participants (57%) self-reported their overall health during 2020 as being about the same as in prior months. However, 36% rated their health as somewhat worse (Fig. 2: Panel 2A). Thematic analyses of open-text responses (Additional file 3) found that participants attributed worsening in health to: (1) a decrease in the amount of exercise or physical activity due to enforced restrictions, closing of gyms/exercise classes and PR programs and a reluctance to attend gyms/group classes due to fear of contracting the virus; (2) deterioration in ILD-related symptoms such as breathlessness that many felt was due to their decrease in exercise; (3) the psychological impact of the pandemic manifesting as stress, anxiety and depression largely associated with a perceived risk or threat of getting COVID but even more so, the possibility of “dying if caught the virus”. Conversely, those who reported an improvement in health attributed it to less socialising and interacting with others that led to being ‘healthier’ with less colds and episodes of being ‘unwell’.

Sixty six percent of participants reported doing less physical activity overall during 2020 compared to before the pandemic, with 42% reported doing a lot less (Fig. 2: Panel 2B).

The majority of respondents (81%) reported that their likelihood of seeking medical assistance during 2020 was about the same compared to before the start of the pandemic. In general, these respondents felt there were no barriers to seeking assistance if and when it was required. However, 14% indicated being less likely to seek assistance with many reporting an avoidance of direct contact with GPs, clinics and hospitals due to their concern regarding the possible risk of contracting the virus at such places, *“…avoiding doctor’s surgery and hospitals especially but also all crowded places” (WA site, female, 73 years).* The 3% who were more likely to seek medical assistance did so due to a deterioration in health that required more frequent monitoring. Participants more likely to seek assistance tended to be younger, female, more likely to have participated in PR, had slightly lower lung function (FVC% predicted) and a lower level of dyspnoea (lower UCSD-SOB score) However, the differences were not statistically significant (Additional file 4).

Access to HCPs during 2020 was rated as ‘good’ by 61% of respondents, but ‘fair to poor’ by 37%. Well managed appointments, availability of HCPs when needed and usefulness of telehealth for scripts and referrals defined ‘good’ access. Others reported difficulties in getting appointments with HCPs and missed assessments (e.g. lung function tests) created a feeling of unease from not being able to monitor their disease. Telehealth was reported by some as less effective and less reassuring to manage deterioration in dyspnoea, or obtain a diagnosis (Fig. 2: Panel 2C).

Access to regular health services such as PR or in-person medical consultations was reduced for 27% of respondents who reported many services stopped running or closed indefinitely. In addition, replacement of in-person interactions with telehealth was not acceptable to all. Issues with access to medications for ILD were reported by 27% of respondents and were related to supply at local pharmacies. During 2020, 66% of respondents reported having a lung function test, 58% had imaging (e.g. CT scan), 85% had blood tests that included monitoring of liver function (for those on antifibrotics) and 19% reported participation in a clinical trial.

Overall, 54% of respondents reported being ‘very satisfied’ with their care during 2020 with a further 42% indicated being ‘satisfied’ or ‘somewhat satisfied’ (Fig. 2: Panel 2D). Higher satisfaction was associated with a reported feeling of being well looked after when needed and having experienced minimal disruptions to care. Less satisfaction was associated with issues of limited access to in person consults, dissatisfaction with telehealth, delays in getting to see GPs, disconnection from services such as PR programs and a feeling that “*more care was provided to those who contracted COVID-19 than people with existing illnesses*”. Those ‘not satisfied at all’ with their care were small in number. On average they were younger, female, not on ILD medications, had a higher level of dyspnoea (USCD-SOB) score and a significantly lower HRQoL (K-BILD score) compared to those more satisfied with their care (Additional file 5). Participants who were cared for exclusively by telehealth were just as likely to be unsatisfied (not satisfied at all or just satisfied) with their care (22% vs 24%) as those cared for through various formats.

Comparison between states showed that the impact on care was similar regardless of site, despite differences in COVID-19 cases and restrictions over time. (Data not shown).

## Discussion

This study examined the impact of the COVID-19 pandemic on the access to care by people with ILD in Australia during 2020. Our findings suggest that the impact on access to care was similar across all four sites regardless of COVID-19 cases and enforced restrictions. Most participants accommodated the rapid transfer from in-person to telehealth, however, telehealth did not always meet the needs of people with ILD especially when unwell. Whilst participants were generally satisfied with the care they received, the delay in fundamental pulmonary assessments and the challenges of remaining physically active, negatively impacted on many participants’ physical and psychological wellbeing.

The pandemic saw telehealth utilisation rapidly expand to provide a safer alternative to in-person consultations for managing the ongoing needs of people with ILD. For some, telehealth was a positive experience that eliminated travel time (especially for those living in regional areas) and provided a ‘safer’ environment for accessing care. However, for others, particularly those who experienced a deterioration in symptoms, the experience was not as positive. Reduced access to HCPs and limited access to in-person consultations made getting care when needed somewhat challenging and frustrating. The reduction of in-person assessments and postponement of key respiratory tests, generated feelings of dissatisfaction, anxiety, and stress about a possible deterioration in lung health.

Contact with respiratory physicians, GPs and nurses played a key role in providing ongoing care, support and information on the pandemic, including support from specialist ILD nurses. However, the majority of consults were conducted by telehealth over the telephone. Similar to other jurisdictions, the Australian government’s public health insurance scheme (Medicare) introduced temporary subsidies to radically expand Australians' access to telehealth. As a result, there was a substantial increase in the use of telehealth during COVID-19, particularly during stricter lockdowns [11, 12]. The extent that participants in our study had a choice in the format of consultations is unclear however, our findings indicated a low utilisation of video conferencing which has the potential to act as an intermediary between in-person and telephone consults. This may be due to factors such as a lack of suitable infrastructure and/or familiarity with using videoconferencing platforms (on both patient and provider end) or patient preference [13]. Emerging from the pandemic, the uncertainty surrounding the future of government funding for Australian telehealth services makes the sustainability of telephone models tentative, highlighting the need for sustainable funding models if telehealth is to become an integrated part of care delivery [14].

A mixed experience of telehealth by people with IPF and other lung conditions has been previously reported. Whilst telemedicine is generally accepted by most, there was a preference for in-person visits [15]. This was particularly true for people with pulmonary disease as it allowed them to undergo pulmonary function tests and have their physician listen to their lungs. Factors associated with a preference for telehealth included male gender, a higher median income and less likely to have comorbidities such as diabetes and hyperlipidemia [15, 16]. Findings from our study emphasise the need for the development of an optimal telehealth model that is tailored to meet individual needs, including patient preference, geographic location, age, gender, socioeconomic background, level of technological practical knowledge and availability of resources, whilst enabling HCPs to monitor and continue providing best care.

Access to key services was another major impact of the pandemic for people with ILD. Although many reported still being able to have lung function assessments and imaging, access to PR programs and support services were significantly impacted. Pandemic-related restrictions saw the closure of exercise hubs and limited opportunities to venture outdoors, forcing many to find alternative means of exercising. In addition, many avoided going outdoors and interacting with others even if permitted, as the fear of contracting the virus and possibly not surviving became overwhelming. In this study, many participants attributed a self-reported worsening in health, particularly lung health, to being less physically active which in turn led to feelings of anxiety, fear, worry and uncertainty regarding their wellbeing. Whilst there is a moderate level of evidence to support the effectiveness of PR in improving functional exercise capacity, dyspneoa and quality of life for people with ILD [17], this study highlighted the self-perceived importance of physical activity and exercise for symptom management and psychological wellbeing in people with ILD. With PR delivered via telehealth showing similar outcomes as centre-based programs, greater access and uptake of such programs may provide a viable option moving forward [18].

The strengths of this study include multi-site recruitment, a high response rate and the use of a questionnaire specifically focused on the health care experience of participants with ILD during the first year of the pandemic. Although the findings may not represent all views and experiences of people with ILD across Australia, participants were recruited from four major tertiary centres that manage patients from large catchment areas incorporating regional areas and smaller Australian states (e.g. Australian Capital Territory). In addition, participants represented a wide range of demographics, ILD types and disease severities. Limitations include the cross-sectional nature of the questionnaire and the potential for recall bias by respondents. Also, we did not ask about the use of remote monitoring devices by participants during this time but this is an approach that could be explored in post-restriction times as a means of providing some reassurance.

## Conclusion

COVID-19 has significantly impacted on the physical and psychological wellbeing of people living with ILD. Whilst there was a general satisfaction with the level of care received, the health care experience for those experiencing deteriorating symptoms was less satisfying, with telehealth not always meeting their needs.

## Supplementary Information

**Additional file 1.** COVID-19 Specific questionnaire. 

**Additional file 2.** Responses to COVID-specific questionnaire.

**Additional file 3.** Thematic analyses from free-text items.

**Additional file 4.** Comparison of participants reporting less likely to seek medical assistance during the pandemic compared to those more or equally as likely to seek care.

**Additional file 5.** Comparison of participants reporting not being satisfied with the care they received during the pandemic compared to those more satisfied with the care received.

## Data Availability

Data generated from this study are not publicly available as consent has not been obtained to share data outside this study but are available from Professor Anne Holland (anne.holland@monash.edu) on reasonable request.
