# Supplementary material for: Understanding the telehealth experience of care by people with ILD during the COVID-19 pandemic: what have we learnt?
Source: BMC Pulm Med. 2023 Apr 6;23:113. doi: 10.1186/s12890-023-02396-6 (PMC10078026; doi:10.1186/s12890-023-02396-6)
Supplement: Supplementary file 1 — Additional file 1. COVID-19 Specific questionnaire. [file 12890_2023_2396_MOESM1_ESM.docx]

**ADDITIONAL FILE 1: COVID-19 SPECIFIC QUESTIONNAIRE**

**IMPACT OF THE COVID-19 PANDEMIC ON THE CARE OF PEOPLE WITH AN**

**INTERSTITIAL LUNG DISEASE - *QUESTIONNAIRE***

The COVID-19 pandemic resulted in a rapid transformation of society and of health services. As we emerge into the post COVD-19 period, it is important for us to evaluate the impact of such changes and what lessons can be learnt.

This questionnaire aims to collect information on the health care experiences of people with an interstitial lung disease during the COVID-19 pandemic period from March to December 2020.

CONSENT: By completing this questionnaire, you are consenting to this research study using the information you provide in publications and presentations in a way that you will not be identified.

**The following questions relate to your experiences in 2020 during the COVID-19 pandemic.**

**Q1. During the pandemic in 2020, did you? (More than one option allowed)**

Self-isolate

Visit family or friends - if permitted during the various stages of COVID-19 restrictions

Visit a supermarket/grocery store - if permitted during the various stages of COVID-19 restrictions

Have contact with a health professional (face-to-face or by video consult)

Undertake any exercise of your own initiative (e.g. go for a walk) - if permitted during the various stages of COVID-19 restrictions

**Q2. During the pandemic in 2020, did you receive any information (verbal or written) about COVID-19 from any health professional?**

No Yes

If YES, please describe

1. Who provided you with the information on COVID-19?

………………………………………………………………………………………………………………………………….

…………………………………………………………………………………………………………………………………..

1. What aspects of COVID-19 did the information cover? *(For example, what to do, who to contact if unwell etc.)*

…………………………………………………………………………………………………………………………………….

……………………………………………………………………………………………………………………………………..

1. To what extent did you find the information you received helpful?

Very helpful Somewhat helpful Not helpful at all

1. Was there any information that was not provided, that you feel would have been helpful to have?

No Yes

1. If YES, please provide details on what information would have been helpful:

…………………………………………………………………………………………………………………………………….

…………………………………………………………………………………………………………………………………….

**Q3. During the pandemic in 2020, did you participate in any exercise or physical activity?**

No Yes

Q3a. If YES, what sort of exercise or physical activity were you doing?

-----------------------------------------------------------------------------------------------------------

Q3b. **How would you rate the amount of physical activity you were doing during the 2020 pandemic compared to before the pandemic?**

A lot less during the pandemic

A little less during the pandemic

About the same amount during the pandemic as before the pandemic

A little more during the pandemic

A lot more during the pandemic

**Q4. How would you rate your overall health during the 2020 pandemic compared to the months prior to the pandemic?**

A lot better during the pandemic

A little better during the pandemic

About the same during the pandemic as before the pandemic

A little worse during the pandemic

A lot worse during the pandemic

Please explain your response

……………………………………………………………………………………………………………………………………………………….

………………………………………………………………………………………………………………………………………………………...

…………………………………………………………………………………………………………………………………………………………

**During the pandemic in 2020, which of the following health professionals did you have contact with regarding your lung health? (More than one option allowed)**

**Q5.**  **GP** No Yes

If YES,

Q5a. Number of consultations with a GP ……………

Q5b. Reason/s for consultation/s (for example you were experiencing more shortness of breath or cough, or needed more medications) or whether this was your regular appointment time:

………………………………………………………………………………………………………………………………………………

……………………………………………………………………………………………………………………………………………….

……………………………………………………………………………………………………………………………………………….

Q5c. How were the consultations conducted? Face-to-face at a clinic, private rooms etc.

By telephone

Video call such as zoom

Other, please specify

-------------------------------------------------------------------------------------------------------------------------------------

Q6. **Respiratory physician**  No Yes

If YES,

Q6a. Number of consultations with a respiratory physician ……………….

Reason/s for consultation/s (for example you were experiencing more shortness of breath or cough, or needed more medications) or whether this was your regular appointment time:

………………………………………………………………………………………………………………………………………………

……………………………………………………………………………………………………………………………………………….

……………………………………………………………………………………………………………………………………………….

Q6b. How were the consultations conducted? Face-to-face at a clinic, private rooms etc.

By telephone

Video call

Other, please specify…………………………………

-------------------------------------------------------------------------------------------------------------------------------------

**Q7. Physiotherapist** No Yes

If YES,

Q7a. Number of consultations with a physiotherapist ……………….

Q7b. Reason/s for consultation/s (for example you were experiencing more shortness of breath or cough) or whether this was your regular appointment time:

………………………………………………………………………………………………………………………………………………

……………………………………………………………………………………………………………………………………………….

…………………………………………………………………………………………………………………………………………………………..

Q7c. How were the consultations conducted? Face-to-face at a clinic, private rooms etc.

By telephone

Video call

Other, please specify…………………………………

------------------------------------------------------------------------------------------------------------------------------------

**Q8.** **Nurse**  No Yes

If YES,

Q8a. Number of consultations with a nurse ……………….

Q8b. Reason/s for consultation/s (for example you were feeling unwell) or whether this was your regular appointment time:

………………………………………………………………………………………………………………………………………………

……………………………………………………………………………………………………………………………………………….

……………………………………………………………………………………………………………………………………………….

Q8c. How were the consultations conducted? Face-to-face at a clinic, private rooms etc.

By telephone

Video call

Other, please specify…………………………………

…………………………………………………………………………………………………………………………………………………………..

**Q9.**  **Other health professionals** No Yes

**If YES, please specify…………………………………………………………………………………**

Q9a. Number of consultations with other specialist ……………….

Q9b. Reason/s for consultation/s or whether this was your regular appointment time:

………………………………………………………………………………………………………………………………………………

……………………………………………………………………………………………………………………………………………….

……………………………………………………………………………………………………………………………………………….

Q9c. How were the consultations conducted? Face-to-face at a clinic, private rooms etc.

By telephone

Video call

Other, please specify…………………………………

-----------------------------------------------------------------------------------------------------------------------------------

**Q10.** I had **no contact** with any health professional regarding my lung health during the 2020 pandemic?

True Not true -- please return to Q5.

**Q11. How would you rate your access to health professionals during the pandemic in 2020?**

Good Fair Not good Poor

Q11a**.** Please provide reason/s for your response

----------------------------------------------------------------------------------------------------------------------------

-----------------------------------------------------------------------------------------------------------------------------

**Q12. During the pandemic in 2020, how likely were you to seek medical assistance compared to before the pandemic?**

Less likely during the pandemic About the same More likely during the pandemic

Q12a**.** Please provide reason/s for your response

----------------------------------------------------------------------------------------------------------------------------

-----------------------------------------------------------------------------------------------------------------------------

**Q13. During the pandemic in 2020, did you have:**

1. An X-ray, CT scan, MRI or any other imaging? No Yes Not sure
2. A blood test? No Yes Not sure

If YES, number of blood tests…………….

If YES, what was the blood test/s for? .................................................................................

1. A lung function test? No Yes Not sure
2. Participated in any clinical trial? No Yes Not sure
3. Had any other test or assessment? No Yes Not sure

If YES, please provide details ………………………………………………………………………………………………..

……………………………………………………………………………………………………………………………………………..

………………………………………………………………………………………………………………………………………………

**Q14. During the pandemic in 2020, did you have a test for COVID-19?**

No Yes Not sure

If YES,

Q14a. How many times were you tested? ………………………………………………………………………………

Q14b Did any of the tests return **positive** for the COVID-19 virus?

No Yes Not sure

**Q15. During the pandemic in 2020, were there any regular health services that you were not able to access or participate in?** (For example: pulmonary rehabilitation, dietician, counselling etc.)

No Yes Not applicable

Q15a**.** If YES, please provide details on what the service/s were and the reason why you could not access them (e.g. pulmonary rehabilitation program at clinic closed)

……………………………………………………………………………………………………………………………………………..

………………………………………………………………………………………………………………………………………………

**…………………………………………………………………………………………………………………………………………**

**Q16. During the pandemic in 2020, did you develop a lung infection?**

No Yes Not sure

**Q17. During the pandemic in 2020, were you been admitted to hospital (that is, you stayed overnight) for any reason?**

No Yes

Q17a. If YES, how many times were you admitted?

……………………………………………………………………………………………………………………………………………

Q17b. What was the main reason for being admitted to hospital?

……………………………………………………………………………………………………………………………………………….

**Q18. During the pandemic in 2020, were you taking any medications for your lung health? *(For example nintedanib (Ofev) or pirfenidone (Esbriet), prednisolone etc.)***

No Yes

If YES,

Q18a. Please describe any issues you may have had with getting access to the medications you were taking during the pandemic (*for example, couldn’t get a prescription from my respiratory physician).*

……………………………………………………………………………………………………………………………………………….

…..………………………………………………………………………………………………………………………………………….

Q18b. What were the consequences (if any) of these issues *(e.g. had to stop taking medications, felt anxious because couldn’t get my script filled)*?

………………………………………………………………………………………………………………………………………………

………………………………………………………………………………………………………………………………………………

……………………………………………………………………………………………………………………………………………….

**Q19. How satisfied have you been with your care during the pandemic in 2020?**

Very satisfied Somewhat satisfied Satisfied Not satisfied at all

Q19a. Please provide reason/s for your response

……………………………………………………………………………………………………………………………………………….

………………………………………………………………………………………………………………………………………….......

_________________________________________________________________________________

**Please complete the following:**

**Contact information:**

**Name: ………………………………………………………………………………………………………………………………………….**

**Address: ……………………………………………………………………………………………………………………………………..**

**Suburb: ………………………………………………………………………………………………………………………………………**

**State: ……………………………… Postcode: …………………………….**

**Email address (if you have one): …………………………………………………………………………………………………**

**Telephone/mobile number ………………………………………………………………………………………………………..**

**Preferred contact method: Mail Email Phone**
