# Supplementary material for: Understanding the telehealth experience of care by people with ILD during the COVID-19 pandemic: what have we learnt?
Source: BMC Pulm Med. 2023 Apr 6;23:113. doi: 10.1186/s12890-023-02396-6 (PMC10078026; doi:10.1186/s12890-023-02396-6)
Supplement: Supplementary file 2 — Additional file 2. Responses to COVID-specific questionnaire. [file 12890_2023_2396_MOESM2_ESM.docx]

**Additional file 2: Responses to COVID-specific questionnaire**

|  | **Total (N=85)**  **n (%)** |
| --- | --- |
| **Q1. Activities undertaken during the pandemic**  Self-isolated  Visited family/friends – when permitted  Visited supermarket/ grocery store – when permitted  Had contact with health professional  Undertaken any exercise of your own initiative | 59 (69)  18 (21)  5 (6)  2 (2)  1 (1) |
| **Q2. Received info about COVID, YES**  Q2a Who provided information on COVID-19? (Open text, more than one option allowed)  GP  Respiratory physician/team  Government health Departments/ media  Nurse  Specialist (e.g. rheumatologist, virologist) | 45 (53)  28 (62)  19 (42)  14 (31)  4 (9)  3 (7) |
| Q2b What aspects of COVID-19 did the information cover?  *- Approaches to keeping safe*  *- General information about virus*  *- Importance of flu and other vaccinations*  *- Wearing masks and hygiene information*  *- Possible symptoms and who to contact if unwell*  *- Advised to isolate if unwell*  *- Advice regarding employment*  *- Caution about risk of contracting virus and possible consequences*  *- Self -monitoring*  *- Importance of caution when interacting with family and wider community due to immunocompromised* | |
| Q2c To what extent did you find the information you received useful?  Very helpful  Somewhat  Not helpful | N=45  27 (60)  18 (40)  0 (0) |
| Q2d Was there any information that was not provided that you feel would have been useful to have? YES  Details if YES,  *- How unwell they could get if they contracted virus*  *- Information on vaccines*  *- Specific risks of COVID on their condition*  *- How to contact lung specialists if needed*  *- Need for government apps to be updated more frequently*  *- How to recognise COVID symptoms over their current condition* | 7 (16) |
| **Q3. During the pandemic have you participated in any exercise or physical activity?** **YES**  Q3a What sort of exercise of physical activity have you been doing? (more than one option allowed)   \| *- Walking* \|  \| \| --- \| --- \| \| *- Exercise classes /gym /weights* \|  \| \| *- Pulmonary rehabilitation* \|  \| \| *- Yoga/Tai chi* \|  \| \| *- Swimming/water classes* \|  \| \| *- Other -golf, gardening, fishing* \|  \|   Q3b How would you rate the amount of physical activity you are doing now compared to what you were doing before the pandemic?  Lot less now  A little less now  About the same now  A little more now  A lot more now | 67 (79)  54 (81)  15 (22)  8 (12)  8 (12)  3 (4)  6 (9)  28 (42)  16 (24)  15 (22)  4 (6)  4 (6) |
| **Q4 How would you rate your overall health during the 2020 pandemic compared to the months prior to the pandemic?**  A little better during the pandemic  About the same during the pandemic as months prior  A little worse during the pandemic  A lot worse during the pandemic | 6 (7)  48 (57)  18 (21)  13 (15) |
| **Q5. During the pandemic, did you have contact with your GP regarding your**  **lung health? YES**  Q5a Number of consultation/s with GP | 56 (66)  Ranged from once to weekly |
| Q5b Reason/s for consultation/s  *- Regular /scheduled appointment*  *- Scripts*  *- Referrals*  *- Blood tests*  *- Lung infection*  *- Shortness of breath*  *- Vaccinations* | |
| Q5c How were the consultations conducted?  FTF at a clinic, private rooms only  Telephone only  Video only  FTF /telephone  Telephone / video  FTF /telephone /video | 11 (20)  12 (21)  0 (0)  30 (54)  1 (2)  1 (2) |
| **Q6. During the pandemic, did you have contact with your RESPIRATORY PHYSICIAN regarding your lung health? YES**  Q6a Number of consultation/s with Respiratory physician | 76 (89)  Range 2 to 9 |
| \| *Q6b Reason/s for consultation/s*  *- Regular appointments*  *- Lung function tests, scans*  *- Appointment with lung transplant specialist* \| \| --- \| \| *- Deterioration in symptoms*  *- Referrals*  *- Script renewal* \| | |
| Q6c How were the consultations conducted?  FTF only  Telephone only  Video only  FTF /telephone  Telephone / video  FTF /telephone /video  FTF/video  FTF/Other  Other | 16 (21)  22 (29)  4 (5)  27 (36)  1 (1.3)  1 (1.3)  1 (1.3)  1 (1.3)  2 (3) |
| **Q7. During the pandemic, did you have contact with a PHYSIOTHERAPIST your lung health? YES** | 15 (18) |
| Q7a Number of consultation/s with physiotherapist | Range 1 to weekly |
| Q7b Reason for consultation/s  *- Regular pulmonary rehabilitation session*  *- Non-pulmonary session*  *- Shortness of breath*  *- Collapsed lung and started using oxygen* | |
| Q7c How were the consultations conducted?  FTF only  Telephone only  Video only  FTF/telephone  Telephone / video  FTF/Video  FTF/other  Other | 7 (47)  0 (0)  1 (7)  3 (13)  0 (0)  1 (7)  1 (0)  2 (13) |
| **Q8. During the pandemic, did you have contact with a NURSE for your lung health? YES**    Q8a Number of consultation/s with nurse | 22 (26)  Range 1 to >8 |
| Q8b Reason for consultation/s  - *Infection—lung, pneumonia*  *- Clinical trial participant*  *- Shortness of breath*  *- Hospitalisation*  - *Blood sample* | |
| Q8c How were the consultations conducted?  FTF only  Telephone only  Video only  FTF /telephone  Telephone / video  FTF /telephone /video  Other | 12 (55)  6 (27)  0 (0)  1 (4)  0 (0)  0 (0)  3 (14) |
| **Q9. Other Healthcare professionals**  FTF only  Telephone only  Video only  FTF /telephone  Telephone / video  FTF /telephone /video  Other | 28 (33)  16 (57)  1 (4)  0 (0)  9 (32)  2 (7)  0 (0)  0 (0) |
| **Q11. How would you rate your access to health professionals during the pandemic?**  Good  Fair  Not good  Poor  Not applicable | 51 (61)  24 (28)  6 (7)  2 (2)  2 (2) |
| **Q12. During the pandemic, how likely are you to seek medical assistance compared to before the pandemic?**  Less likely now  About same  More likely now | 14 (16)  65 (77)  6 (7) |
| **Q13a** Had an X-ray, CT scan, MRI or any other imaging? YES | 56 (66) |
| **Q13b** Had a blood test? YES | 72 (85) |
| **Q13c** Had a lung function test? YES | 56 (66) |
| **Q13d** Participated in a clinical trial? YES | 16 (19) |
| **Q13e** Had any other test/ assessment? YES | 25 (29) |
| **Q14. During the pandemic did you have a test for COVID-19?** **YES**  Q14a How many times were you tested?  Q14b Number of positive COVID tests | 35 (41)  Range 1 -7  0 (0) |
| **Q15. During the pandemic, were there any regular health services that you were not able to access or participate in? YES**  Q15a Services not able to access/participate in:  - *Pulmonary rehabilitation programs*  *- Lung function testing*  *- Face-to-face appointments with respiratory physician*  *- Appointments with other specialists e.g. dietician, psychologist*  *- Lung biopsy* | 23 (27) |
| **Q16. During the pandemic did you develop a lung infection? YES** | 19 (22) |
| **Q17. During the pandemic, have you been admitted to hospital for any reason? YES**  Q17a Number of times admitted to hospital  Q17b Main reason for hospital admission  - *Infections*  *- Shortness of breath/low oxygen levels*  *- Lung biopsy*  *- Kidney related procedures*  *- Other* *e.g. surgery* | 24 (28)  Range 1-7 times  8 (33)  5 (21)  3 (13)  3 (13)  5 (21) |
| **Q18 During the pandemic, were you taking any medications for your lung health? (For example nintedanib (Ofev) or pirfenidone (Esbriet)) YES**  Medication type   \| *- Methotrexate only* \| \| --- \| \| *- Mycophenolate only* \| \| *- Methotrexate and prednisolone* \| \| *- Nintedanib only* \| \| *- Pirfenidone only* \| \| *- Nintedanib and pirfenidone* \| \| *- Prednisolone only* \| \| *- Nintedanib and prednisolone* \| \| *- Mycophenolate and prednisolone* \| \| *- Rituximab* \| | 65 (77)  4 (6)  23 (37)  1 (2)  10 (16)  7 (11)  1 (2)  12 (19)  1 (2)  3 (5)  1 (2) |
| Q18a Details of any issues you may have had with getting access to the medications you were taking during the pandemic   - *Supply at local pharmacy* - *Script related -dispensing rule* | 12 (27%) |
| Q18b Consequences (if any) issues with access to medications  - *Delay in starting treatment*  *- Stopped medications for short period* | |
| **Q19 How satisfied have you been with your care during the pandemic (2020)?**  Very satisfied  Somewhat satisfied  Satisfied  Not satisfied at all | 46 (54)  19 (22)  17 (20)  3 (4) |
