# Supplementary material for: Understanding the telehealth experience of care by people with ILD during the COVID-19 pandemic: what have we learnt?
Source: BMC Pulm Med. 2023 Apr 6;23:113. doi: 10.1186/s12890-023-02396-6 (PMC10078026; doi:10.1186/s12890-023-02396-6)
Supplement: Supplementary file 3 — Additional file 3. Thematic analyses from free-text items. [file 12890_2023_2396_MOESM3_ESM.docx]

**Additional file 3: Thematic ANALYSES from free-text items**

**Q4 How would you rate your overall health during the 2020 pandemic compared to the months prior to the pandemic?**

| **RATING** | **SUMMARY OF RESPONSES** | **THEMES** |
| --- | --- | --- |
| A little better during pandemic | - Able to continue exercising  - Less colds and being unwell due to reduced social interactions and going out  -Less work-related stress  - Healthier eating habits – not going out to eat | - No colds, or unwell during 2020 as less socialising and interaction with others - Decrease in amount of exercise or physical activity able to   do due to:  -restrictions  -closing of gyms  -reluctance to attend gyms etc. due to risk of getting virus   - Deterioration in symptoms associated with lung conditions—breathlessness, cough, fatigue, weight loss etc.   --primarily attributed to decrease in exercise   - Psychological impact—stress, depression, anxiety   -- increased stress associated with risk/threat of COVID and possibility of “dying if caught virus”   - Isolation |
| About the same during the pandemic as months prior | - Self isolation was nothing new  - Lung health remained stable, no self-reported changes in symptoms  - Little change to routines  - Able to continue exercise as usual |  |
| A little worse during the pandemic | - Self-reported increase in symptoms – cough, breathlessness, fatigue  - Feelings of anxiety and depression  - Worried about catching COVID and would they die  - Reduction in physical activity  - Closure of gyms, pulmonary rehabilitation programs  - Increased stress due to COVID  - Not able to go out  - Difficulty working with a mask on - more breathless |  |
| A lot worse during the pandemic | - Unable to exercise and felt lung condition got worse  - Felt risks and threats of pandemic stressful and difficult to deal with  - Felt everything got harder to manage  - Diagnosed with cancer  - Didn’t exercise or go out due to concerns about catching COVID  - Increased stress form work, isolation and depression  - Lung condition flared up |  |

**Q11. How would you rate your access to health professionals during the pandemic in 2020?**

| **RATING** | **SUMMARY OF RESPONSES** | **THEMES** |
| --- | --- | --- |
| Good | - Still able to have consults be telephone and blood tests and spirometry  - Found telehealth ‘fabulous’- much easier  - HPs always helpful, access was good  - HPs provided advice and answered questions  - Scripts and referrals provided by phone  - Minimal impact of COVID – able to continue normal access to HPs  - Appointments arranged without delay  - Lung clinic staff helpful in giving advice and making appointments for other consults | **Good access**  - Appointments well managed  - Telehealth worked well for scripts and referrals  - HPs made time for patients when needed and provided information  **Fair**  - Appointments harder to get  - Missed scans, LFT created a *feeling of unease* as couldn’t monitor symptoms  - Telehealth –not ideal  -- “especially when having breathing difficulties”  *-- less effective, less reassuring*  *-- unable to provide a diagnosis*  **Not good/Poor**  **-** Possibility of missing significant health issues due to lack of monitoring  - Lack of information about who to contact and what to do |
| Fair | - Delay in getting first COVID vaccination  - Delay in getting to see lung specialist  - Long waiting time for appointments with HPs  - No in-person appointments with lung specialist and no lung function testing – felt uneasy about not knowing where lung health was at  - Avoided face-to-face contact with HPs  - Not able to be monitored via telehealth  -Telephone consults not ideal, less reassuring without physical exam  - Fair given the circumstances  - Felt not monitored as frequently as would have liked given had a lung condition |  |
| Not good | - Appointments cancelled if during lockdown period  - High possibility of missing significant health issues due to lack of tests and monitoring  - No helpful information provided unless participant pushed for it  - Access to HPs through public health system was very disappointing |  |
| Poor | - Telehealth appointments could not provide results to assessments  - Unsure as to who to contact in time of need |  |

**Q12. During the pandemic in 2020, how likely were you to seek medical assistance compared to before the pandemic?**

| **RATING** | **SUMMARY OF RESPONSES** | **THEMES** |
| --- | --- | --- |
| Less likely now | - Avoided contact with clinics/hospitals/crowded places unless necessary due to exposure risks  - Restrictions closed travelling to appointments in city  - Local GP practice not seeing anyone face-to-face  - Aware of pressure HPs under so avoided contact unless necessary | - Avoided contact/interaction with clinics/GPs/hospitals due to concerns regarding risk of contracting virus - Consideration of strain HPs were under and didn’t want to un-necessarily add to this - No need to seek assistance—disease stable, had no issues - Sought assistance if and when required– no barriers to accessing HPs   Deterioration in health   - Deterioration in health and new health conditions necessitated more frequent contact and monitoring of health issues |
| About the same | - No change  - Telehealth was a flexible option  - Saw HP when needed  - Continued regular appointments with GP and specialists  - Tried to maintain routine check-ups to avoid complications  - No issues/condition stable so no need to seek more assistance  - Always have good access and contact  - All HPs made it easy to contact them in a safe manner |  |
| More likely now | - Deterioration in health required more frequent contact and monitoring  - Diagnosed with other health conditions that required treatment and hospitalisation |  |

**Q19. How satisfied have you been with your care during the pandemic in 2020?**

| **RATING** | **SUMMARY OF RESPONSES** | **THEMES** |
| --- | --- | --- |
| Very satisfied | - HPs always available –provided confidence  - Found care by GP and specialist to be excellent  - Minimal disruption to normal care whether face-to-face or telephone  - Able to have regular appointments by phone  - No issues with scripts | - Respondents were generally satisfied with care received - Satisfaction associated with:   - confidence provided by availability of HPs and care provided  - able to have regular appointments to keep monitoring health   - Less satisfaction associated with:   - less positive experience with telehealth  - delay in diagnosis  - feeling that people with COVID were provided with more care than those with pre-existing conditions |
| Somewhat satisfied | - Preference for face-to-face contact  - Lack of in-person contact difficult when suffering mental health issues  - Generally satisfied with care  - Felt disconnected due to lack of respiratory monitoring  - Given circumstances, happy with care received |  |
| Satisfied | - Felt more care was provided to those with COVID than those with pre-existing illnesses  - Phone consults not a great experience- felt it didn’t achieve as much  - Able to get assistance and medications without any issue  - Delay in getting oxygen supply |  |
| Not satisfied at all | - Lack of information provided by HPs on services available during this time  - Interruption to treatment resulted in deterioration of lung condition, now require double lung transplant. Major impact on financial security |  |
