# Supplementary material for: Understanding the telehealth experience of care by people with ILD during the COVID-19 pandemic: what have we learnt?
Source: BMC Pulm Med. 2023 Apr 6;23:113. doi: 10.1186/s12890-023-02396-6 (PMC10078026; doi:10.1186/s12890-023-02396-6)
Supplement: Supplementary file 4 — Additional file 4. Comparison of participants reporting less likely to seek medical assistance during the pandemic compared to those more or equally as likely to seek care. [file 12890_2023_2396_MOESM4_ESM.docx]

**ADDITIONAL FILE 4: Comparison of participants reporting less likely to seek medical assistance during the pandemic compared to those more or equally as likely to seek care.**

| **Characteristic*** | **Less likely to seek medical assistance during the pandemic** | **More likely or equally as likely to seek medical assistance during pandemic** |
| --- | --- | --- |
| **Age**, *years (mean ± SD)* | N=13  77.15 ± 10.40 | N=69  65.30 ± 10.97 |
| **Gender,** female | N=14  7 (50%) | N=70  45 (64%) |
| **ILD type**  CTD-ILD  HP  IPF  NSIP  Sarcoidosis  Scleroderma  Unclassifiable ILD  Other | N=13  1 (8%)  1 (8%)  3 (23%)  3 (23%)  2 (17%)  0 (0%)  2 (15%)  1 (8%) | N=69  18 (26%)  5 (7%)  17 (25%)  11 (16%)  5 (7%)  1 (1%)  5 (7%)  7 (9%) |
| **Smoking status**  Never  Former  Current | N=13  4 (31%)  7 (58%)  1 (8%) | N=67  25 (37%)  41 (61%)  1 (1.5%) |
| **ILD medications** use, *yes* | N=15  11 (73%) | N=75  57 (76%) |
| **Oxygen use** – currently, *yes* | N=13  2 (15%) | N=68  12 (18%) |
| **Pulmonary rehabilitation in last 12 months,** *yes* | N=13  2 (15%) | N=67  15 (22%) |
| **BMI**, kg/m2 *(mean ± SD)* | N=13  29.71 ± 6.90 | N=59  29.65 ± 5.76 |
| **6MWT**,** m  *(mean ± SD)* | N=11  440.73 ± 112.44 | N=50  463.84 ± 137.33 |
| **FVC %predicted **** *(mean ± SD)* | N=13  79.15 ± 18.89 | N=56  74.53 ± 16.16 |
| **DLCO %predicted**** *(mean ± SD)* | N=13  57.43 ± 14.30 | N=58  58.26 ± 17.61 |
| **Main comorbidities**  Autoimmune rheumatological disease  Heart disease  Diabetes mellitus  Non haematological cancer  Chronic kidney disease | N=6  2 (33%)  1 (17%)  3 (50%)  0 (0%)  0 (0%) | N=30  10 (33%)  6 (20%)  8 (27%)  5 (17%)  1 (3%) |
| **K-BILD transformed score**, *(mean ± SD)* | N=14  57.80 ± 14.84 | N=73  55.35 ± 12.67 |
| **UCSD -SOB score**, *(mean ± SD)* | N=14  46.93 ±25.29 | N=71  42.90 ± 25.07 |

* Total numbers vary depending on availability of data in medical record

CTD-ILD – *Connective tissue disease-ILD*; HP – *hypersensitive pneumonitis*; IPF – *idiopathic pulmonary fibrosis*; NSIP – *Nonspecific interstitial pneumonia;*

6MWT – *6 minute walking test;* FVC% pred *-* *forced vital capacity % predicted;* DLCO%pred *- diffusing capacity for carbon monoxide %predicted*; K-BILD - *The King’s Brief Interstitial Lung Disease;* USCD-SOB- *The University of California San Diego Shortness of Breath*

** Refers to last result prior to date when face-to-face clinic consultations changed to telehealth
