# Supplementary material for: Understanding the telehealth experience of care by people with ILD during the COVID-19 pandemic: what have we learnt?
Source: BMC Pulm Med. 2023 Apr 6;23:113. doi: 10.1186/s12890-023-02396-6 (PMC10078026; doi:10.1186/s12890-023-02396-6)
Supplement: Supplementary file 5 — Additional file 5. Comparison of participants reporting not being satisfied with the care they received during the pandemic compared to those more satisfied with the care received. [file 12890_2023_2396_MOESM5_ESM.docx]

**ADDITIONAL FILE 5: Comparison of participants reporting not being satisfied with the care they received during the pandemic compared to those more satisfied with the care received.**

| **Characteristic*** | **Not satisfied at all with care during pandemic in 2020** | **Satisfied, somewhat or very satisfied with care during the pandemic in 2020** |
| --- | --- | --- |
| **Age**, *years (mean ± SD)* | N=3  57.96 ± 8.87 | N=79  66.37 ± 10.97 |
| **Gender,** female | N=3  2 (67%) | N=82  51 (62%) |
| **ILD type**  CTD-ILD  HP  IPF  NSIP  Sarcoidosis  Scleroderma  Unclassifiable ILD  Other | N=3  0 (0%)  0 (0%)  1 (33%)  0 (0%)  1 (33%)  0 (0%)  0 (0%)  1 (33%) | N=79  19 (24%)  6 (8%)  19 (24%)  14 (18%)  6 (8%)  1 (1%)  7 (9%)  7 (9%) |
| **Smoking status**  Never  Former  Current | N=2  0 (0%)  1 (50%)  1 (50%) | N=78  29 (37%)  48 (62%)  1 (1%) |
| **ILD medications** use, *yes* | N=3  1 (33%) | N=87  67 (77%) |
| **Oxygen use** – currently, *yes* | N=3  0 (0%) | N=78  14 (18%) |
| **Pulmonary rehabilitation in last 12 months**, *yes* | N=3  0 (0%) | N=77  17 (22%) |
| **BMI,** kg/m2 *(mean ± SD)* | N=3  32.73 ± 2.40 | N=69  29.53 ± 6.00 |
| **6MWT**, m  [last result prior to clinic closure]  *(mean ± SD)* | N=2  393.00 ± 249.90 | N=58  461.93 ± 130.26 |
| **FVC %pred** *(mean ± SD)*  [last result prior to clinic closure] | N=2  88.20 ± 15.27 | N=67  75.02 ± 16.65 |
| **DLCO %pred** *(mean ± SD)*  [last result prior to clinic closure] | N=3  56.53 ± 5.32 | N=68  58.17 ± 17.31 |
| **Main comorbidities**  Autoimmune rheumatological disease  Heart disease  Diabetes mellitus  Non haematological cancer  Chronic kidney disease | N=2  0 (0%)  0 (0%)  2 (100%)  0 (0%)  0 (0%) | N=34  12(35%)  7 (21%)  9 (26%)  5 (15%)  1 (3%) |
| **K-BILD transformed score**, *(mean ± SD)* | N=3  36.77 ± 6.29 | N=84**  56.42 ± 12.66 |
| **UCSD -SOB score**, *(mean ± SD)* | N=3  62.00 ± 21.28 | N=83  43.25 ± 25.04 |

* Total numbers vary depending on availability of data in medical records; **p=0.01 (Rank sum test)
